# Bridging the service gap in cognitive behavioral therapy from a user perspective: Findings from a web‐based survey in Japan

**DOI:** 10.1002/pcn5.70240

**Published:** 2025-11-11

**Authors:** Tadashi Haraguchi

**Affiliations:** ^1^ Health Service Center Utsunomiya University Utsunomiya Japan

**Keywords:** cognitive behavioral therapy, health literacy, health services accessibility, information dissemination, patient Preference

## Abstract

**Aim:**

The primary aim was to quantify public awareness, experience, and delivery‑format preferences regarding cognitive behavioral therapy (CBT) in Japan. The secondary aim was to identify user‑related factors associated with CBT literacy (awareness or experience), including information‐seeking behavior and prior service use.

**Methods:**

A cross‑sectional web survey of 518 Japanese residents aged 15–79 years was conducted in August 2024. Awareness and experience of CBT, preferred delivery formats, mental‑health service use, mindfulness recognition, and Certified Public Psychologist (CPP) awareness were assessed. A binary logistic regression examined factors linked to CBT awareness/experience.

**Results:**

Only 13.1% of respondents reported awareness of or experience with CBT, while 62.2% had never heard of it. Face‐to‐face CBT with physicians was preferred by 59.3%, compared with 8.9% favoring application‑based delivery. Awareness of mindfulness (odds ratio [OR] = 13.68, 95% confidence interval [CI] 6.83–27.40), CPP awareness (OR = 5.48, 2.48–12.10), and past mental‑health service use (OR = 3.17, 1.52–6.58) were independently associated with CBT literacy; age, gender, and self‑rated mental health were not significantly associated factors.

**Conclusion:**

Public awareness of CBT in Japan remains limited despite more than a decade of insurance coverage. Engagement with mental‐health services, exposure to mindfulness concepts, and familiarity with CPPs appear to be associated with greater CBT literacy. Most users prefer face‐to‐face delivery, suggesting eed to expand trained providers while gradually integrating digital options.

## Graphical abstract

A nationwide web survey of 518 Japanese residents quantified public awareness, experience, and preferred delivery formats of cognitive behavioral therapy (CBT). Of the respondents, 87% had little or no knowledge of CBT, while 59% preferred physician‐led face‐to‐face delivery. Past mental‐health service use (OR = 3.17), mindfulness exposure (OR = 13.68), and Certified Public Psychologist recognition (OR  = 5.48) independently predicted CBT literacy.

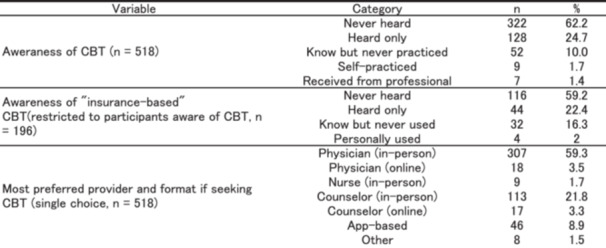

## INTRODUCTION

Cognitive behavioral therapy (CBT) is among the most evidence‐based psychological treatments and has demonstrated strong efficacy in managing mental disorders, such as depression and anxiety.^1^, ^2^ In addition to its clinical effectiveness, CBT has also been shown to promote stress management and enhance mental well‐being in non‐clinical populations.^1^, ^3^, ^4^, ^5^ Despite its broad applicability and robust empirical support, a service gap of CBT persists in many countries: many individuals who could benefit from CBT do not receive it.^6^ In this study, “service gap” denotes the mismatch between the need for CBT and actual receipt of CBT services (access/uptake); “treatment gap” refers to the broader shortfall between those meeting criteria for mental disorders and those receiving any evidence‐based care^6^; and “research–practice gap” describes the distance between established evidence and routine delivery.^7^ The focus here is on user‐side factors related to the CBT service gap in Japan.

To address this gap, several countries have implemented national strategies to expand access to CBT. Notably, the United Kingdom′s Improving Access to Psychological Therapies (IAPT) program introduced a structured training scheme for psychological therapists within the National Health Service (NHS), enabling large‐scale delivery of CBT across NHS care pathways.^8^, ^9^ While this approach has expanded service availability and facilitated outcome monitoring, challenges in dissemination and accessibility remain, suggesting that effective interventions alone are insufficient to ensure widespread use.^10^ The program started in 2008; in 2023, it was renamed “NHS Talking Therapies.”

In Japan, CBT was first included in the National Health Insurance system for the treatment of depression in 2010, and its coverage expanded in 2016 to include anxiety disorders and related conditions.^11^, ^12^ However, nationwide data analyses have shown that the implementation of CBT under insurance schemes remains extremely limited. Hayashi et al. revealed substantial regional disparities and a low volume of CBT sessions despite coverage.^13^ Haraguchi et al. found that the 2016 policy expansion did not lead to a significant increase in CBT provisions.^11^ In line with these findings, a previous report indicated that CBT was delivered by only a minority of providers, citing barriers such as workforce shortages, time constraints, and institutional limitations.^12^ While these studies provide insight into the public insurance‐based system, the extent to which CBT is delivered in private or non‐insurance settings remains unclear due to a lack of systematic data. This lack of visibility complicates efforts to evaluate access and develop comprehensive dissemination strategies.

Recognizing these challenges, Japan′s Health and Global Policy Institute (HGPI) issued a set of policy recommendations emphasizing the need to promote the dissemination of CBT and CBT‐based support methods across healthcare, education, welfare, and occupational settings.^14^ The report highlighted the importance of developing comprehensive policy frameworks, establishing multi‐professional training systems, integrating digital CBT into service delivery, and implementing incentive mechanisms to encourage wider adoption.

Most previous Japanese studies on the dissemination of CBT have concentrated on provider‐side perspectives, such as regulatory frameworks, reimbursement systems, and workforce development.^11^, ^12^, ^13^ While this research has elucidated critical structural and institutional barriers, user‐side factors, including public awareness, preferences, and expectations, remain poorly understood in the Japanese context. For instance, there is limited empirical evidence regarding the general population′s recognition of CBT, their experience with it, or their preferred modes of delivery. A better understanding of these demand‐side elements is crucial for promoting service uptake. Despite the growing international emphasis on patient‐centered and user‐informed policy designs,^15^, ^16^, ^17^, ^18^, ^19^ Japan lacks systematic studies that address the perspectives of potential users. This imbalance may contribute to a persistent gap between service provision and utilization, thereby limiting the effectiveness of supply‐driven dissemination strategies.

The expansion of digital CBT has been supported by growing evidence of its effectiveness in both clinical and non‐clinical settings.^5^, ^20^, ^21^, ^22^, ^23^, ^24^ A range of modalities, including internet‐based programs, mobile apps, and blended care models, have been evaluated, and the outcomes are generally positive.^5^ However, there is a limited understanding of how potential users perceive these modalities or what formats they prefer. Preference for the face‐to‐face versus online delivery, concerns about therapeutic alliances, and individual differences in acceptability have been highlighted as important but underexplored factors.^25^, ^26^ In addition, mental health professionals have varied opinions on the appropriateness and challenges of digital CBT, indicating that clinical endorsement cannot be assumed.^27^, ^28^ At the same time, growing reliance on digital tools places greater responsibility on individuals to identify trustworthy resources, often without sufficient guidance or support.

In today′s decentralized information environment, individuals are expected to actively seek, assess, and select mental health resources on their own. However, this process is complicated by information asymmetry, a long‐standing issue in healthcare, whereby providers possess substantially more knowledge than users.^29^ This asymmetry is particularly problematic in digital mental health,^30^ where the quality and trustworthiness of online information vary widely. Evidence‐based sources coexist with commercially driven content, unregulated mobile apps, AI‐generated advice, and user‐submitted commentary.^31^, ^32^, ^33^ Without centralized evaluation mechanisms or professional guidance, individuals, especially those with low digital literacy, must navigate a fragmented and often confusing information landscape.^31^ These challenges hinder informed decision‐making and may contribute to disparities in access to appropriate care. ^34^

Japan introduced the Certified Public Psychologist (CPP) system in 2017 as Japan′s first nationally licensed psychologist qualification.^12^, ^35^ Although CBT is included in their training curriculum, CPPs are currently not permitted to deliver CBT under the national health insurance system. Consequently, insured CBT must be provided by physicians or trained nurses, many of whom face substantial workload constraints. Previous reports have identified this structural limitation as a key barrier to wider CBT implementation and have proposed expanding the role of CPPs to meet unmet needs.^2^, ^12^ Compared to the UK′s NHS Talking Therapies (formerly IAPT) program, which trained non‐physician therapists to deliver CBT at scale,^8^, ^9^, ^10^ Japan′s system offers a less clearly defined role for CPPs.^35^ While some experts have proposed that CPPs could contribute to evidence‐based practices such as CBT,^12^, ^35^ public awareness of their role and expectations regarding their involvement remain largely unexplored.

The literature on public awareness, experience, and preferences for CBT is scarce; to the author′s knowledge, only a Japanese outpatient survey (in Chiba Prefecture) and a Portuguese general‐population study of prospective acceptability/preferences (Unified‐Protocol CBT) have been published.^36^, ^37^ Consequently, evidence on user‐side awareness, experience, and preferences for CBT in the general population is limited and remains unclear. Given its public salience as a third‐wave CBT‐related construct, awareness and experience of mindfulness was included as a user‐side correlate of CBT literacy.

The primary aim was to quantify public awareness, experience, and preferred delivery formats regarding CBT in Japan through a web‐based survey. The secondary aim was to identify user‐related factors associated with CBT literacy (awareness or experience) to clarify the user–provider service gap and inform strategies for improving the dissemination of CBT nationwide.

## METHODS

### Study Design and Participants

This cross‐sectional web‐based survey was conducted in August 2024. Reporting aligns with Strengthening the Reporting of Observational Studies in Epidemiology (STROBE) and Checklist for Reporting Results of Internet E‐Surveys (CHERRIES); participation metrics are summarized in Supplementary Table S‐R1. Participants were recruited from Macromill′s Japan online panel (approximately 10 million members). Enrollment followed first‐come, first‐served within vendor‐managed age‐stratified quotas (non‐random). Survey invitations and distribution were managed by the vendor. Per vendor policy, the numbers of invitations, views/opens, and entries were not disclosed to investigators. Accordingly, the number of completed questionnaires (*n* = 518) was reported; completion was defined as submission of all mandatory items on the final page. Conventional response rates were not calculated because denominators were unavailable, in line with CHERRIES. A completed STROBE checklist indicating item‐level manuscript locations is provided in the Supplement.

### Survey Procedures

Single submission per panel account was enforced by the vendor, and mandatory items were required for completion. Participants received reward points managed by Macromill; no additional incentives were provided by the investigator. Basic demographics (age, gender) and socio‐demographic attributes (occupation, residence, marital status, presence of children, student category, household and personal income) were not self‐reported. These variables were obtained from the panel registration database and linked to responses, with no personally identifying information provided to the investigator. Desktop and mobile devices were permitted. Inclusion criterion was Japanese residents aged 15–79 years (vendor panel age range), excluding junior high school students.

### Survey Instrument

The questionnaire evaluated six domains: (1) awareness, personal experience, and preferred delivery formats for CBT (e.g., in‐person vs. online, physician‐led vs. counselor‐led), (2) history of mental health service use, (3) awareness and experience of mindfulness, (4) recognition of CPP qualification and expectations regarding CBT, (5) initial action when concerned about one′s own mental health, and (6) information‐seeking behavior. Most questions were single‐choice, and the subjective mental health status was assessed using a five‐point Likert scale (1 = *poor* to 5 = *good*), and mental health service use history permitted multiple responses to capture the diversity of past experiences. The full questionnaire (exact item wording and anchors) is provided in Supporting Information S1: Appendix S1.

### Statistical Analysis

Descriptive statistics summarized CBT awareness, experience, and preferences. Two‐group comparisons between the CBT‐unaware/name‐only and CBT‐aware/experienced groups were conducted using Pearson′s chi‐square tests; results are presented in Supporting Information S2: Table S1.

To examine the factors associated with CBT awareness/experience, a binary logistic regression model was fit:

CBT Awareness/experience _logit_ = β₀ + β₁ (Gender) + β₂ (Age) + β₃ (Subjective mental health status) + β₄ (Mental‐health service use) + β₅ (Initial response behavior) + β₆ (Mindfulness awareness/experience) + β₇ (CPP awareness/experience) + ε

Outcome coding: CBT awareness/experience (aware/experienced [include “know the term but never practiced”/self‐practice/received from a professional] = 1, unaware/name‐only = 0).

Explanatory variables were as follows: gender (*female* = 1, *male* = 0); age (continuous); subjective mental health status (*poor/somewhat poor* = 1, *otherwise* = 0); mental‐health service use in the past three years (*any* = 1, *none* = 0); initial response behavior when first concerned about own mental health (*help‐seeking/information seeking* = 1, *self‐distraction/inaction* = 0); mindfulness awareness/experience (*aware/experienced [include ‘know the term but never practiced’/self‐practiced/received from a professional]* = 1, *unaware/name only* = 0); CPP awareness/experience (*aware/experienced* = 1, *aware/name‐only* = 0), including those who had received counseling but were unsure whether the counselor was a CPP (*n* = 4).

Covariate selection was prespecified to reflect user‐side factors most proximal to CBT literacy while preserving parsimony given the number of events (≈60–70). Income variables had high “unknown/no‐answer” rates, and occupation comprised small categories and sparse‐data bias; these SES variables were excluded. Primary internet source variables were excluded from the model because they lacked a clear pre‐specified role. Adjusted odds ratios (ORs) with 95% confidence intervals (CIs) are reported. Model fit was evaluated using the omnibus model χ² and the Hosmer–Lemeshow test Nagelkerke R² was also reported. Statistical significance was set at *P* < 0.05. Analyses were performed using IBM SPSS Statistics (version 29.0).

### Ethical Considerations

This study was approved by the Ethics Committee of Utsunomiya University (approval no. H24‐0043). The approved research title was “Survey on Information‐Seeking and Decision‐Making Related to Mental Health.” All data were collected anonymously, and electronic informed consent was obtained from all participants prior to the start of the survey.

## RESULTS

### Participant Characteristics and Mental‐Health‐Related Variables

A total of 518 valid responses were obtained: 87 respondents each from the teens (aged15‐19), 20s, 30s, 40s, and 50s, and 83 respondents aged 60–79 years. Completed questionnaires totaled *n* = 518; vendor‐level denominators (invitations, views/entries) were not disclosed, so conventional response rates could not been calculated (Supporting Information S2: Table S1). Of the 518 respondents, 239 were men (46.1%) and 279 were women (53.9%). The mean age was 40.6 years (SD = 17.3; range = 15–79; Table 1). Table 1 also summarizes mental‐health‐related variables. Self‐rated mental health varied: 18.1% reported “good,” whereas 23.2% reported “somewhat poor” or “poor.” In terms of service use, 19.0% had accessed at least one mental‐health service in the past three years, most commonly in‐person psychiatric consultation (13.1%). Initial coping behaviors when first concerned about mental health included distraction (32.4%), doing nothing (26.1%), and online information seeking (22.8%). Regarding information sources, Google (60.2%) and Yahoo! JAPAN (25.9%) were the most frequently used search engines, whereas social‐media platforms accounted for ≤4%. For mindfulness, 34.4% had heard of the concept, but only 5.8% had ever practiced it.

**Table 1:** Demographic characteristics, mental health status, and help‐seeking behavior (*n* = 518).

Mean age (years)	40.6 (SD = 17.3), range: 15–79		
Gender	men = 239 (46.1%), women = 279 (53.9)		
Variable	Category	*n*	%
Subjective mental health (past month)	Good	94	18.1
	Somewhat good	134	25.9
	Fair	170	32.8
	Somewhat poor	86	16.6
	Poor	34	6.6
Experience of mental health services (past 3 years)	No use	419	80.9
	Psychiatrist (in‐person)	68	13.1
	Psychiatrist (online)	3	0.6
	Counselor (in‐person)	28	5.4
	Counselor (online)	10	1.9
	Mental health app	14	2.7
Initial response behavior when concerned about mental health	Distract self (e.g., hobbies, exercise, etc.)	168	32.4
	Do nothing	135	26.1
	Search online or on social media	118	22.8
	Talk with family or friends	49	9.5
	Consult a physician	30	5.8
	Others	8	1.5
	Consult books	5	1
	Consult a psychologist or counselor	5	1
Most preferred internet platform when searching for mental health	Google	312	60.2
	Yahoo!	134	25.9
	Instagram	22	4.2
	YouTube	21	4.1
	TikTok	10	1.9
	X (formerly Twitter)	9	1.7
	Others	7	1.4
	ChatGPT	3	0.6
	Facebook	0	0
Awareness of mindfulness	Unaware (never heard)	340	65.6
	Name‐only	114	22
	Know but never practiced	34	6.6
	Self‐practiced	21	4.1
	Received from a professional	9	1.7

### Awareness and Experience of CBT

Among 518 respondents, 62.2% had never heard of CBT, 24.7% had heard the term only, 10.0% knew about it but had never practiced it, 1.7% had self‐practiced, and 1.4% had received CBT from a professional (Table 2). Thus, only 13.1% (*n* = 68) demonstrated meaningful awareness or experience.

**Table 2:** Awareness and experience of CBT, and preferences and expectations for its delivery format.

Variable	Category	*n*	%
Awareness and experience of CBT (*n* = 518)	Never heard	322	62.2
	Heard only	128	24.7
	Know but never practiced	52	10.0
	Self‐practiced	9	1.7
	Received from a professional	7	1.4
Awareness of “insurance‐based” CBT(restricted to participants aware of CBT, *n* = 196)	Never heard	116	59.2
	Heard only	44	22.4
	Know but never used	32	16.3
	Personally used	4	2
Most preferred provider and format if seeking CBT (single choice, *n* = 518)	Physician (in‐person)	307	59.3
	Physician (online)	18	3.5
	Nurse (in‐person)	9	1.7
	Counselor (in‐person)	113	21.8
	Counselor (online)	17	3.3
	App‐based	46	8.9
	Other	8	1.5

*Note*: Awareness of insurance‐based CBT was assessed only among participants who reported any awareness of CBT (i.e., those who did not select “Never heard” in the first item; *n* = 196).

Abbreviation: CBT, Cognitive Behavioral Therapy.

### Two‐group comparisons

Two‐group comparisons between the unaware/name‐only and aware/experienced groups are summarized in Supporting Information S2: Table S1. Significant differences were observed for mental health service use (χ²[1] = 39.57, *P* < 0.001), mindfulness awareness (χ²[1] = 127.86, *P* < 0.001), and CPP awareness (χ²[1] = 81.61, *P* < 0.001). Age showed a borderline overall association (χ²[5] = 11.07, *P* = 0.050). No significant differences were found for gender (*P* = 0.922), subjective mental health (*P* = 0.488), or initial response behavior (*P* = 0.122).

### Binary logistic regression analysis

Binary logistic regression was used to examine factors associated with CBT awareness and experience. The model was significant (Omnibusχ²[7] = 127.545, *P* < 0.001) with acceptable fit (Hosmer–Lemeshow χ²(8) = 11.754, *P* = 0.163; Nagelkerke *R²* = 0.404). As shown in Table 4, these three variables independently increased the odds of CBT awareness and experience.

1) Mindfulness awareness*** (OR = 13.68, 95% CI: [6.83, 27.40], *P* < 0.001)
2) CPP awareness*** (OR = 5.48, 95% CI: [2.48, 12.10], *P* < 0.001)
3) Use of mental‐health services** in the past three years (OR = 3.17, 95% CI: [1.52, 6.58], *P* = 0.002)

In brief, individuals who were aware of mindfulness, familiar with the CPP system, and had used any mental health service in the past three years were approximately 13.7‐fold, 5.5‐fold, and 3.2‐fold more likely, respectively, to be aware of or have experience with CBT.

Other factors, including gender, age, subjective mental health status, and initial response behavior style, were not significantly associated (all *P* > 0.05).

### Preferences for CBT Delivery

As shown in Tables 2, 59.3% of participants preferred to receive CBT in‐person from a physician, whereas 21.8% preferred face‐to‐face delivery with a counselor—a ratio of almost 3:1. Online formats attracted little interest (3.5% for a physician and 3.3% for a counselor), and only 8.9% favored a mobile‐app option. These figures indicate a clear preference for face‐to‐face delivery over digital format.

### Awareness and Expectations of Certified Public Psychologists (CPPs)

As shown in Table 3, awareness of the CPP system was limited: 73.9% of respondents had never heard of it, 15.8% had heard only the name, while a further 7.5% knew about it but had never received services, and <3% had ever consulted a CPP.

**Table 3:** Awareness, experience, and expectations regarding Certified Public Psychologists (CPPs) in Japan (*n* = 518).

Section	Response option	*n*	%
Awareness and Experience with CPPs	Did not know (first time hearing it)	383	73.9
	Know the name only	82	15.8
	Know about it, but never received services	39	7.5
	Personally received counseling from one	10	1.9
	Received counseling, but unsure if licensed	4	0.8
Expectations Toward CPPs Providing Insurance‐Based CBT	Expect	141	27.2
	Somewhat expect	218	42.1
	Neutral	126	24.3
	Somewhat do not expect	12	2.3
	Do not expect	21	4.1

*Note*: Item assessed under a hypothetical scenario in which CPPs would be allowed to provide CBT under the national health insurance in Japan.

Abbreviation: CBT, Cognitive Behavioral Therapy.

Despite this low awareness, a majority supported the idea of CBT being delivered by CPPs under national health insurance system: 27.2% answered “yes” and 42.1% “somewhat yes,” totaling 69.3% in favor.

## DISCUSSION

### Implications of Limited Public Awareness of CBT

This study investigated the awareness, experience, and preferences of the general population regarding CBT in Japan. The findings demonstrated that public recognition and experience of CBT remain notably low, with only 13.1% of respondents reporting that they had either heard of or experienced CBT (Table 2). These results are consistent with those of previous studies using national insurance claims data, which show that CBT utilization in Japan has remained limited despite policy efforts to expand its coverage.^11^, ^13^

Importantly, participants who rated their own mental health as “poor” or “somewhat poor” were not significantly more likely to be aware or have experience with CBT compared with those reporting better self‐rated mental health (Table 4). This suggests a critical access gap, wherein evidence‐based psychological interventions do not effectively reach individuals who may benefit the most from them. Such a gap may be partially explained by informational asymmetry in the healthcare field,^29^, ^33^ whereby professionals possess substantially more knowledge than users, leading to an unqual access to services.

**Table 4:** Logistic regression model: Factors associated with CBT awareness or experience (*N* = 518).

Variable	B	SE	OR	95% CI	*p* value
Constant	−3.277	0.585	0.04	–	<0.001***
Age (per year)	0.003	0.010	1.00	0.98–1.02	0.800
Sex (*female* = 1)	−0.087	0.334	0.92	0.48–1.76	0.795
Subjective mental health (*poor/somewhat poor* = 1)	−0.159	0.382	0.85	0.40–1.80	0.676
Mental‐health service use in the past 3 years (*yes* = 1)	1.152	0.374	3.17	1.52–6.58	0.002**
Initial response behavior (*help‐seeking/information‐seeking* = 1)	0.241	0.345	1.27	0.65–2.50	0.484
Mindfulness awareness (*know/practiced* = 1)	2.616	0.354	13.68	6.83–27.40	<0.001***
CPP awareness (*aware/used* = 1)†	1.702	0.406	5.48	2.48–12.10	<0.001***

*Note*: Reference categories: male, good/average mental health, no mental health service use, self‐distraction/inaction coping, unaware/name only of mindfulness, unaware/name only of Certified Public Psychologists (CPPs). OR = odds ratio. Model fit: Omnibusχ²(7) = 127.545, *P* < 0.001; Nagelkerke R² = 0.404; Hosmer–Lemeshow χ²(8) = 11.754, *P* = 0.163.

Abbreviations: B, unstandardized coefficient; CBT, Cognitive Behavioral Therapy; CI, confidence interval; CPP, Certified Public Psychologist; SE, standard error; SNS, social networking service.

** *P* < 0.01,

*** *P* < 0.001,

^†^ CPP awareness coded 1 includes “used but unsure” (*n* = 4).

This mismatch between need and awareness underscores the limitations of the current dissemination strategies in Japan. Expanding the availability of CBT under health insurance would be insufficient if those most in need remain unaware of its existence or effectiveness. To address this, policy initiatives must consider not only structural supply factors (e.g., health insurance) but also user‐side elements, such as mental health literacy and access to credible information sources.^30^, ^33^

### Role of Mental Health Service Use and Professional Awareness

The logistic regression analysis identified three factors associated with CBT awareness and experience (Table 4): prior mental health service use, mindfulness awareness, and awareness of CPPs. This finding suggests that engagement with services or psychological concepts appears to provide entry points into evidence‐based information.

First, participants who had used mental health services in the past three years were more likely to be aware of or have experience with CBT. This finding aligns with reports that help‐seeking behaviors often predict higher mental health literacy.^6^, ^38^, ^39^ In Japan, Kanehara et al. reported that the leading reason for not accessing mental healthcare was low perceived need and that structural barriers, such as not knowing where to obtain help, outweighed attitudinal barriers.^40^ Taken together, these observations point to a gap in public awareness that may limit the adoption of CBT. Our data imply that contact with mental health services offers a key opportunity for introducing CBT to potential users.

Second, mindfulness awareness was significantly associated with CBT awareness and experience. Although this survey did not measure the participants′ understanding of mindfulness, both clinical and societal factors may underlie this relationship. Clinically, mindfulness‐based interventions are classified as the third wave of CBT.^41^ Mindfulness and CBT share overlapping frameworks, such as awareness of automatic thoughts and emotional regulation, with CBT techniques such as cognitive restructuring.^41^, ^42^ Socially, mindfulness has reached the public through books, media, smartphone applications, and workplace wellness programs in non‐clinical contexts.^43^ Thus, even people who have never used mental health services may have encountered CBT‐related ideas (e.g., thought monitoring and stress management) via mindfulness content.^44^ These observations suggest that exposure to mindfulness in either clinical or nonclinical settings could introduce individuals to CBT. This interpretation is consistent with our a priori rationale for including mindfulness: as a third‐wave, CBT‐related construct with high public salience, mindfulness awareness/experience functions as a pragmatic user‐side correlate (or gateway) of CBT literacy.

Third, awareness of CPPs, Japan′s national license for psychological professionals, was also significantly associated with the awareness and experience of CBT. To our knowledge, this is the first study to quantify the public recognition of CPPs and examine their association with CBT literacy. Although CPPs cannot currently provide CBT under the national health insurance system, they work in schools, employee assistance programs, and community settings.^35^ In these contexts, they may incorporate CBT principles into routine psychological support, thereby familiarizing users with CBT concepts. People who recognize CPPs may, therefore, possess broader mental health literacy, including evidence‐based therapies. These findings imply that indirect contact with CPPs outside the insurance system can broaden the public awareness of CBT.

Taken together, the three factors of recent service use, recognition of CPPs, and mindfulness awareness showed independent links to CBT knowledge or experience. This finding indicates that both clinical contact and wider cultural exposure can broaden the public′s familiarity with evidence‐based therapies. However, our cross‐sectional design cannot establish causality; future longitudinal or experimental work should examine whether introducing mindfulness content or enhancing access to psychological professionals directly increases CBT literacy.

### Public Preferences and the Importance of Face‐to‐Face Delivery

User preference for the CBT delivery format emerged as a key finding. Regarding CBT delivery formats, most respondents preferred face‐to‐face sessions with physicians or counselors; only 8.9% preferred an app, and 15.6% selected video sessions (Table 2). This preference underscores the value placed on interpersonal contact in therapeutic settings; however, it poses scalability and accessibility challenges, because Japan lacks sufficient CBT‐trained therapists, and access differs markedly across regions.^11^, ^13^

Since the coronavirus disease (COVID‐19) pandemic in 2019, online and digital mental health services have expended rapidly^20^, ^23^, ^28^, ^45^ A growing body of research has demonstrated the efficacy of internet‐based CBT and digital delivery formats from the provider perspective,^5^, ^20^, ^22^, ^26^ yet prior studies on user preferences have shown mixed results, with some emphasizing reduced stigma and greater convenience, whereas others stress the value of human interaction and therapeutic alliance in face‐to‐face settings.^25^, ^27^, ^46^ Although our data show that users may not fully endorse digital CBT as a replacement for in‐person care, almost one in ten participants favored an app‐based option, suggesting the scope for technology‐assisted interventions to complement traditional care.

Differences in attitudes suggest that digital CBT, although effective, is not acceptable to all users. Researchers have proposed blended models that integrate digital resources with therapist support.^26^, ^47^ These findings imply that acceptability and trust are critical factors in the successful dissemination of CBT, and must be addressed alongside clinical efficacy.^11^, ^13^ Digital services can narrow this gap. However, delivery formats must match the users′ preferences to earn acceptance.^17^, ^18^, ^19^ Future dissemination strategies should therefore balance clinical efficacy and credibility with a shared user‐provider perspective to ensure that CBT reaches those who need it the most.

### Awareness of Certified Public Psychologists and Their Role in CBT Dissemination

This study is the first to examine the public awareness of Japan′s CPP qualification and its association with CBT awareness. Although CPPs have been licensed psychological professionals since 2017,^35^ their recognition remains low (Table 2). Nevertheless, most respondents supported allowing CPPs to provide CBT under the national health insurance system (Table 3).

Currently, however, CPPs cannot bill for CBT under national insurance, and its provision is largely restricted to physicians. Insurance data indicate that CBT sessions have hardly increased, even after reimbursable diagnoses were expanded.^11^, ^13^ In contrast, the UK′s NHS Talking Therapies (formerly IAPT) demonstrates how structured training and service integration can scale CBT; therapists are trained in CBT and embedded within the NHS care pathways.^8^, ^10^ Japan lacks such coordinated training and deployment systems. However, our data suggest that the public expects CPPs to be potential providers of CBT under national insurance.

Although the awareness of CPPs remains modest, their potential contribution to CBT delivery is considerable. Policy reforms should focus on expanding training pipelines, extend insurance reimbursements, and embed CPPs more firmly within care networks. Clarifying and strengthening these roles could broaden access to evidence‐based psychotherapy, as demonstrated by international models.

### Information Access and the Future Role of Generative Artificial Intelligence

Although search engines remain the dominant tool for mental health information seeking, we found no association between the type of online service used and CBT awareness (Tables 1 and 4). Thus, access to general information does not automatically expose users to evidence‐based interventions. When feeling mentally unwell, most participants either distracted themselves or did nothing, whereas a minority actively searched for information or consulted professionals. Among information seekers, search engines vastly outnumbered books, social media, and generative AI tools. However, previous studies have shown that the quality of online mental health content varies considerably,^38^, ^39^ which can reinforce literacy gaps. Our findings confirm that even those who use digital tools may not encounter evidence‐based treatment. This gap reflects information asymmetry, wherein expert knowledge does not reliably reach the public.^48^ Mental health literacy, which is defined as the ability to locate, understand, and apply evidence‐based information, is therefore crucial for converting online engagement into evidence‐based treatment.^39^, ^49^

The rise of generative AI platforms, such as ChatGPT, introduce new possibilities. They can deliver tailored, on‐demand content that might enhance mental health literacy at scale.^31^, ^32^ However, the risks include biased outputs, misinformation, and user overreliance.^34^ As AI becomes embedded in digital health ecosystems,^50^ future studies should critically evaluate its impact on information accuracy, user trust, and behavioral changes, including its potential to reduce regional and socioeconomic disparities in accessibility.

In summary, internet tools are widely used for seeking mental health information, but the pathway from information seeking to recognizing evidence‐based treatments, such as CBT, remains fragmented. Improving the quality of digital mental health contents is necessary but not sufficient; users also need the skills to discern and act on reliable guidance. A rigorous evaluation of generative‐AI applications should form part of future policy and research agendas aimed at narrowing information asymmetries and expanding equitable access to psychological services such as CBT.

### Limitations

This study has several limitations. First, because provider‐level invitation, view, and entry counts were not disclosed, conventional response rates could not be calculated, and nonresponse bias could not be quantified. The first‐come, first‐served panel sampling method also limits generalizability beyond online panel users. Second, the online panel sample may have underrepresented individuals without access to the internet, thereby restricting generalizability. Third, its cross‐sectional design precludes causal inference. Fourth, self‐reported data may have been influenced by recall or social desirability biases. Fifth, personal or family psychiatric history was not collected because it is not part of the standard panel profile and would have required an optional paid module; therefore, residual confounding by this factor cannot be ruled out. Finally, the number of participants with actual CBT experience was limited, potentially reducing the statistical power of the subgroup analyses.

## CONCLUSION

Public awareness and experience with CBT in Japan remain limited, even among those with perceived mental health concerns. CBT awareness was associated with prior mental health service use, mindfulness awareness, and knowledge of CPPs. While face‐to‐face delivery was preferred, some interest in digital formats was observed. These findings underscore the persistent service gap and highlight the importance of expanding user‐centered, multimodal dissemination strategies for CBT.

Future research should employ random sampling and include currently treated populations to enhance generalizability. Qualitative studies could clarify barriers to CBT and informational needs. As artificial intelligence becomes more prevalent in health information seeking, studies should also assess its impact on treatment awareness and decision‐making. Further evaluation of digital and hybrid CBT delivery models is essential for improving access.

## AUTHOR CONTRIBUTIONS

Tadashi Haraguchi conceived the study, developed the questionnaire, performed the data analysis, and wrote the manuscript.

## CONFLICT OF INTEREST STATEMENT

The author declares no conflicts of interest.

## ETHICS APPROVAL STATEMENT

The study protocol was approved by the Ethics Committee of Utsunomiya University (approval no. H24‐0043).

## PATIENT CONSENT STATEMENT

Electronic informed consent was obtained from all participants.

## CLINICAL TRIAL REGISTRATION

Not applicable.

## Supporting information

PCN Reports supplementary tables PCNR‐2025‐0172 20251019.

CBT survey draft appendix 20250713.

## Data Availability

The data that support the findings of this study are available upon reasonable request from the corresponding author. The data are not publicly available due to privacy and ethical restrictions. The de‐identified dataset generated and analyzed during the current study cannot be shared publicly available due to contractual and ethical restrictions with Macromill, Inc. Aggregated results and analysis code are available from the corresponding author upon reasonable request and with approval from both the Ethics Committee of Utsunomiya University and Macromill, Inc.
