# Supplementary material for: Bridging the service gap in cognitive behavioral therapy from a user perspective: Findings from a web‐based survey in Japan
Source: PCN Rep. 2025 Nov 11;4(4):e70240. doi: 10.1002/pcn5.70240 (PMC12603917; doi:10.1002/pcn5.70240)
Supplement: Supplementary file 1 — PCN Reports supplementary tables PCNR‐2025‐0172 20251019. [file PCN5-4-e70240-s001.docx]

****P* < .001.*P*-values are from chi-square tests. CBT, Cognitive Behavioral Therapy
