# Supplementary material for: Bridging the service gap in cognitive behavioral therapy from a user perspective: Findings from a web‐based survey in Japan
Source: PCN Rep. 2025 Nov 11;4(4):e70240. doi: 10.1002/pcn5.70240 (PMC12603917; doi:10.1002/pcn5.70240)
Supplement: Supplementary file 2 — CBT survey draft appendix 20250713. [file PCN5-4-e70240-s002.docx]

**Survey Questionnaire**

1. Please choose one statement that best describes your mental health condition during the past month.

(a) Good

(b) Fairly good

(c) Average

(d) Fairly poor

(e) Poor

1. In the past three years, have you used any “mental-health services,” such as medical consultations, counseling, or smartphone apps? (Check all that apply.)

(a) None

(b) In-person physician visits

(c) Online or telephone consultations with a physician

(d) In-person counseling with a psychologist or counselor

(e) Online or telephone counseling with a psychologist or counselor

(f) Mental health apps (smartphones)

(g) Other services (please specify _______)

1. When you feel concerned about your own mental health (e.g., stress, insomnia, low mood, and lack of motivation), what is the first thing you usually do? (Select one.)　If you have never experienced such concerns, answer as you would if they have occurred.

(a) Do nothing

(b) Look up information in books.

(c) Search the internet

(d) Talk with family or friends

(e) Consult a physician

(f) Consult a psychologist or counselor

(g) Other (please specify _________)

1. If you look for information about your mental health on the internet or social media, which platform do you use the most? (Select one.)

(a) Google

(b) Yahoo

(c) X (formerly Twitter).

(d) Instagram

(e) TikTok

(f) Facebook

(g) Other (please specify _________)

1. How familiar are you with “mindfulness” or “mindfulness meditation”? (Select one.)

(a) Do not know (first hearing)

(b) Have heard only of the term

(c) I know about it but have never practiced it

(d) I practiced it myself, but not with a professional.

(e) Have received mindfulness instruction from a professional (physician, counselor, etc.)

1. How familiar are you with psychotherapy known as “Cognitive Behavioral Therapy (CBT)”? (Select one.)

(a) Do not know (first hearing)

(b) Have heard only of the term

(c) I know about it but have never practiced it

(d) I practiced it myself, but not with a professional.

(e) Having received CBT from a professional

1. Health-insurance–covered CBT for depression or anxiety disorders is available in Japan. Which statements apply to you? (Select one.)

(a) Did not know about this (first hearing)

(b) Have heard about it to some extent

(c) Knew about it but never received it

(d) Has it been received personally?

1. If you were to receive CBT for depression or anxiety disorders, which delivery method would you prefer? (Select one.) CBT improves mental health by changing thoughts and behaviors. Its effectiveness has been scientifically demonstrated not only for clinical conditions but also for stress management among students and workers.

(a) In person with a physician

(b) Online with a physician

(c) In person with a nurse

(d) Online with a nurse

(e) In person with a psychologist or counselor

(f) Online communication with a psychologist or counselor

(g) Mental health application

(h) Other (please specify _________)

1. Regarding the national qualification “Certified Public Psychologist” (CPP)—Japan’s first state-licensed psychology profession—choose the statement that best describes you. (Select one.) Note: This differs from private certifications such as “clinical psychologist.”

(a) Do not know (first hearing)

(b) Only known by name

(c) I know about it but have never used its services.

(d) Have personally used its services

(e) Have received counseling but did not know whether the counselor was a CPP.

1. Currently, Certified Public Psychologists (CPPs) cannot provide Cognitive Behavioral Therapy (CBT) under national health insurance. To what extent do you support permitting CPPs to deliver insurance-covered CBT to patients with depression or anxiety disorders? (Select one.) CPPs are state-licensed professionals established under the Certified Public Psychologists Act (2017). They provide psychological support across healthcare, welfare, education, and other fields.

(a) Support

(b) somewhat support

(c) Neutral

(d) Somewhat oppose

(e) Oppose
